# Traumatic Foreign Body into the Face: Case Report and Literature Review

**DOI:** 10.1155/2017/3487386

**Published:** 2017-01-03

**Authors:** Maysa Nogueira de Barros Melo, Lidyane Nunes Pantoja, Sara Juliana de Abreu de Vasconcellos, Viviane Almeida Sarmento, Christiano Sampaio Queiroz

**Affiliations:** ^1^Federal University of Bahia, Salvador, BA, Brazil; ^2^Department of Diagnostics and Therapeutics, Dentistry School, The Federal University of Bahia, Araújo Pinho Avenue, No. 62, 40110-150 Canela, Salvador, BA, Brazil

## Abstract

This paper describes a case of mouth opening limitation, secondary to a facial trauma by cutting-piercing instrument, whose fragments had not been diagnosed in the immediate posttrauma care. Description of an unusual surgical maneuver and a literature review are presented.

## 1. Introduction

Punctate and incised/piercing wounds are described as injuries that occur because of perforating and cutting/piercing instruments such as knives and splinters, which violate cutaneous or mucosal barriers [1]. Foreign bodies or their fragments—resulting from fracture of these instruments—although often found in the oral cavity and maxillofacial region [2], are rarely reported in the literature [3].

These lesions may represent a challenging situation for the oral maxillofacial surgeon due to many factors, such as object size, difficult access, and the proximity of the foreign body to vital structures [4].

Occasionally, foreign bodies may remain impacted for some time, causing persistent and distressing symptoms [5]. Some of them may remain in situ for clinical reasons [6] and removing them could bring more harm than benefits. Most of them, however, are removed before the onset of complications, remarkably infection [7].

It is essential to find exactly where the foreign body is located before its removal [4]. It is therefore important to perform imaging examination, as plain radiographs, computed tomography (CT scans), magnetic resonance imaging (MRI), and ultrasound, depending on the location and composition of the foreign body [8, 9]. These should be recent at the time of surgery, because of the migration risk to adjacent areas [10, 11].

Treatment of punctate and cutting-incised wounds on the face includes suturing, bone fracture reductions and fixation, and, in severe cases, facial reconstruction [12].

This paper describes a case of limited mouth opening, secondary to facial trauma by cutting-incised object (glass), whose fragments had not been diagnosed in the immediate posttrauma care, remaining in the region of the infratemporal fossa. A brief literature review is also presented.

## 2. Literature Review

Foreign bodies are often found in facial wound but rarely reported in the literature [3]. Some authors believe that the head is the body region most frequently affected by trauma, and facial involvement is very common due to the face exposure [13].

According to Sastry et al. (1995) [14], the lodgment of foreign body in an area like infratemporal fossa is quite rare and only few cases have been reported in the literature so far. Wulkan et al. (2005) [15] also report complications associated with the foreign body removing due to its critical structures: excessive hemorrhage, infection, pain, swelling, and trismus.

There are some indications to foreign body removing, listed in Table 1, and foreign body most common sites according to the literature are found in Table 2.

## 3. Case Report

Male patient, brunette skin, 28 years old, attended the outpatient clinic of the Department of Oral & Maxillofacial Surgery of Santo Antônio Hospital (affiliated to Federal University of Bahia), complaining about progressive mouth opening limitation after assault by a cutting-piercing instrument, 35 days before.

**Figure 1:**
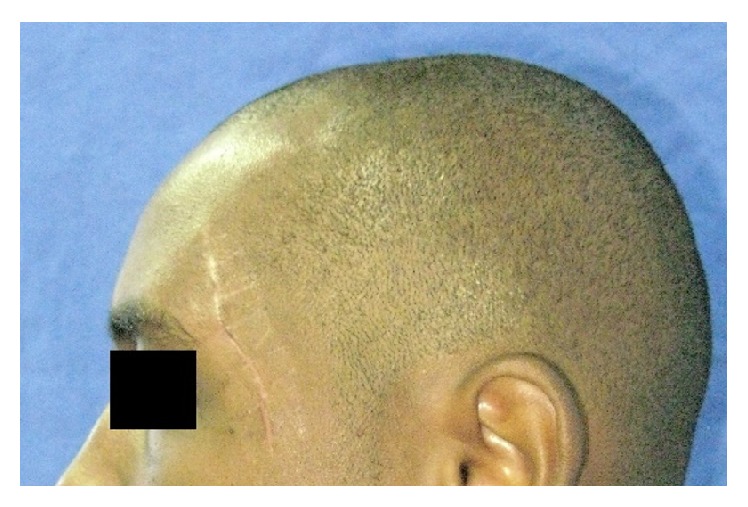
Clinical preoperative aspect (scar on the temporal region).

**Figure 2:**
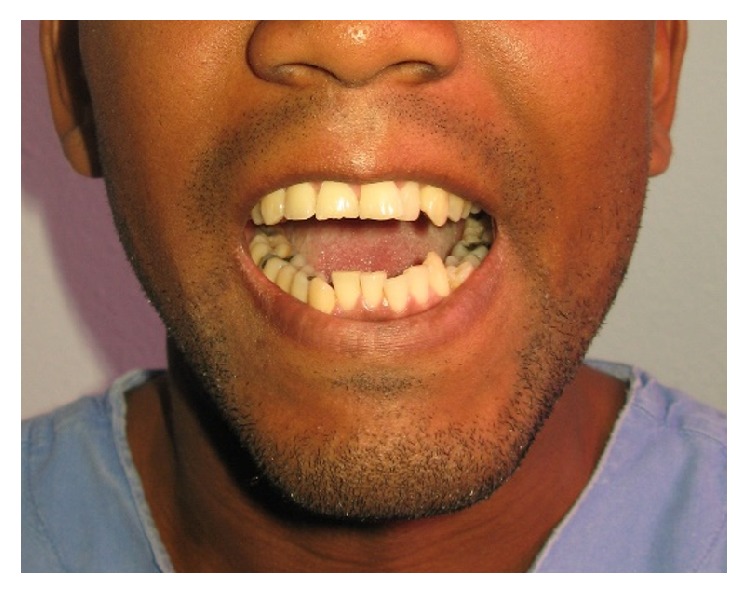
Opening mouth limitation.

Clinical examination revealed a hypochromic linear scar on the left temporal region, corresponding to the aggression site (Figure 1). Suture was performed in immediate posttrauma care, without imaging exams and the patient did not remember the type of object that stroked him. Unilateral paralysis of the scalp muscle was noticed, configuring frontal branch injury of the seventh cranial nerve pair. No signs of facial fractures were observed. The mouth opening—the main complaint—was restricted, with interincisal distance of approximately 24 mm (Figure 2). When asked about tetanus prophylaxis, the patient said he had been vaccinated in less than a 10-year period.

**Figure 3:**
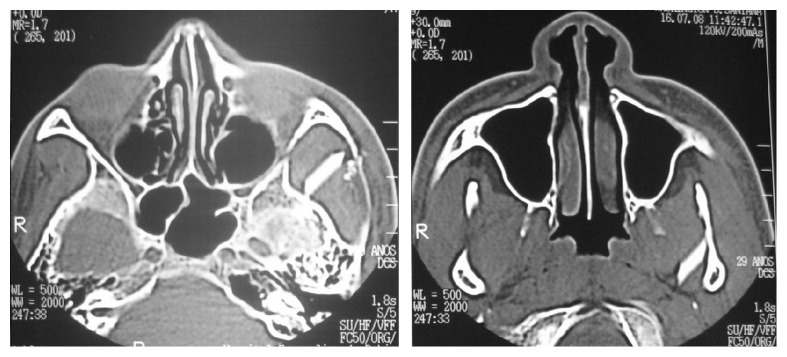
Computed tomography scan (hyperdense images medially to left zygomatic arch near to mandibular ramus).

The computed tomography (CT) scan showed two rectangular hyperdense images: one medially to the zygomatic arch and the other one medially to the mandibular ramus (Figure 3). We removed the foreign bodies under general anesthesia via preauricular access with temporal extension. We chose this access over an approach by the entry scar, because the object fragments were distant from the entrance site.

**Figure 4:**
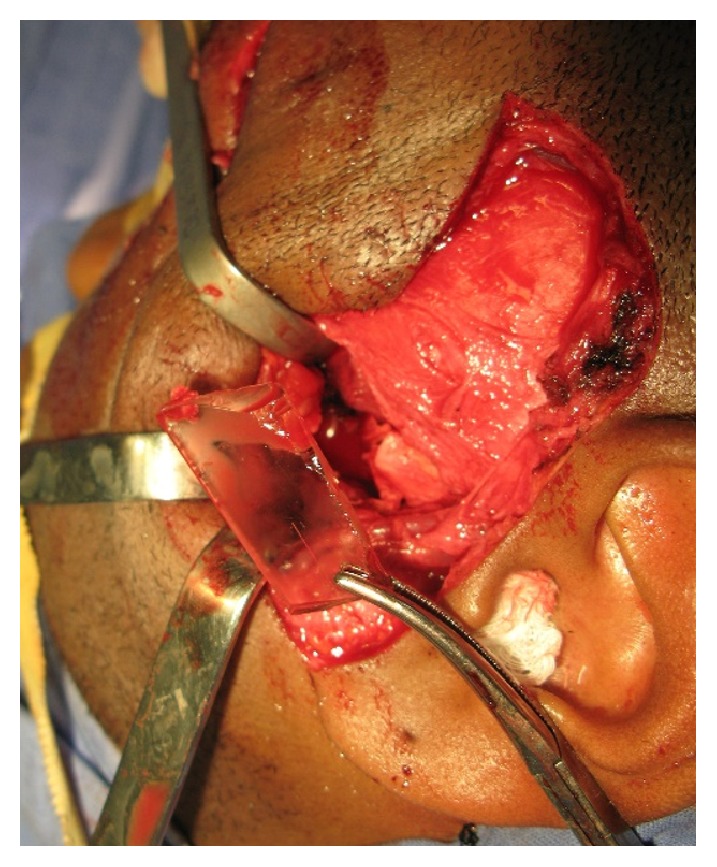
First foreign body removal.

During the intraoperative exploration, there was difficulty in locating the object fragments. A zygomatic arch ostectomy was made then, after which a colorless fragment of hard consistency and smooth surface—possibly glass—was palpable and carefully removed (Figure 4). Further exploration allowed us to locate and remove the second fragment.

We performed osteosynthesis of the zygomatic arch with two stainless steel wires. Irrigation and aspiration of the operative site with posterior suture of the tissue planes finalized the surgical procedure.

**Figure 5:**
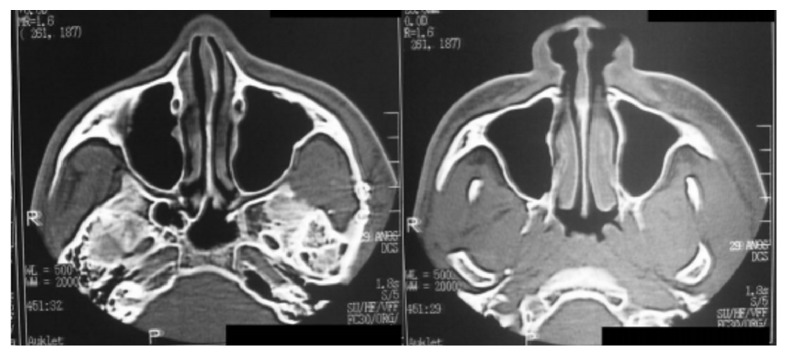
Postoperative computed tomography images.

**Figure 6:**
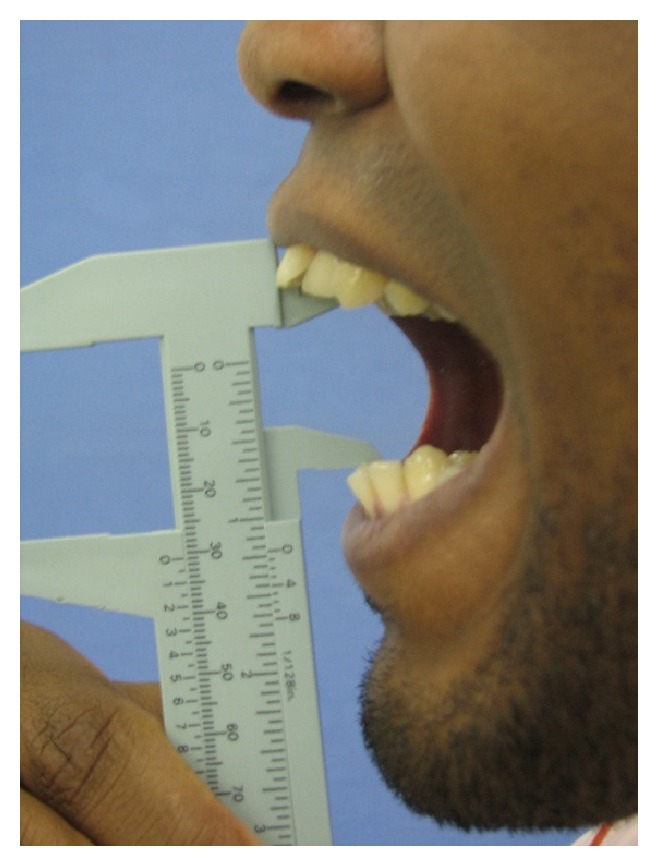
Mouth opening at 45th postoperative day.

The first postsurgical review followed a week later. Mouth opening had a slight improvement (28 mm of interincisal distance) and CT showed infratemporal fossa without foreign body fragments (Figure 5). Physiotherapy was initiated two weeks after the surgery. Forty-five days after the operation, we observed a mouth opening of 31 mm (Figure 6).

## 4. Discussion

Punctate and cutting-incised wounds can be considered one of the most devastating attacks because of the emotional consequences and the possibility of deformity [19]. This case exemplifies the deforming character of these lesions, illustrated by the presence of extensive hypertrophic scar in the left temporal region.

That patient belongs to gender and age group (20–39 years) most affected by facial trauma, and the etiology of the trauma that attacked him (interpersonal violence) fits as the most frequent [20].

The prompt removal of foreign bodies from the intimacy of body parts may not occur due to a misdiagnosis or absence of symptoms [7]. That is what happened in the reported case: the glass fragments in the infratemporal fossa were not found in immediate posttrauma care and the patient sought treatment only when he realized the limitation of mouth opening. The delay in treating these cases can lead to definitive limitations or even death [20]. Unlike in more commonly observed cases [9], no infection was found.

As recommended by Shinohara et al. [20], the following steps were made: access, foreign body removal, exploration of the wound, irrigation, and suturing, in addition to certifying about tetanus prophylaxis [21] and use of antimicrobials [16].

Metallic objects and glass splinters as foreign bodies as in this case are more frequent and well tolerated by the body, while organic materials cause more inflammation and can lead to serious complications [18, 22]. Metal objects are most commonly readily diagnosed by physical examination or conventional imaging studies. Glass fragments, however, may have diagnosis delayed until the appearance of clinical complications such as skin lesions, cellulite [23], or granuloma [24]. Mouth opening limitation was the complication observed in this case. Not performing imaging tests on the patient's initial care was a major factor to the misdiagnosis. Knowing the object that caused the injury is very important to choose the type of imaging test to be requested. Glass fragments would hardly be properly diagnosed by plain radiographs [17].

The limitation of mouth opening after trauma with cutting-piercing instruments is a sign that may suggest infection by* Clostridium tetani* [25, 26]. Thus, the maxillofacial surgeon should consider this possibility if local factors justifying this sign cannot be found. In the reported case, glass fragments were this cause, probably due to fibrosis of the injured musculature, inflammatory reaction to foreign bodies, or even foreign bodies acting as physical barriers to mandible movement.

The treatment of cutting-piercing wound victims with retention of foreign bodies in the maxillofacial region should be often conducted by a multidisciplinary team including maxillofacial surgeons, radiologists, otolaryngologists, ophthalmologists, and vascular surgeons, due to the possibility of profuse bleeding during or after removal of the foreign body [20]. In this case, the surgery was uneventful, but it did require caution in handling the foreign body, since the sharp borders of the glass could cause vessel damage during removal. The chosen approach was closer to the foreign body and avoided esthetical losses on the scar area. The same approach was described by Sajad et al. in 2011 [4] to solve a similar clinical situation. According to Wulkan et al. 2005 [15], little is known about the best strategy for removing foreign bodies in infratemporal regions.

## 5. Conclusion

Foreign bodies misdiagnosed causes complicated medical problems and sometimes the surgical operations are necessary. When they are in infratemporal fossa—a closed anatomic space that includes neurovascular vital structures—it is important to provide a safe and effective solution, as showed in the reported case.

On trismus complaint cases, they should be included in the differential diagnosis, especially in patients with recent past history of trauma.

## Figures and Tables

**Table 1:** Indications to remove facial foreign bodies.

Remove	Do not remove
Organic [16]	Inorganic [16]
Freely palpable [16]	Posterior orbit (organic or inorganic) [16]
Anterior orbit (organic or inorganic) [16]	Proximity to vital structures [15]
Reactivity, heavy contamination, or toxicity [17]	Absence of imaging exams [17]
Intra-articular location, persistent pain [17]	Risk of iatrogenic injury [4]
Infection, psychological distress [17]	Absence of symptoms [17]
Impairment of mechanical function [17]	Unknown precise location [17]

**Table 2:** Facial foreign bodies common sites.

Authors	Region
Perumall et al. 2014 [16]	Intraorbital/mandible/frontal bone
Wulkan et al. 2005 [15]	Infratemporal fossa
Vikram et al. 2012 [17]	Zygomatic
Sajad et al. 2011 [4]	Infratemporal fossa
Moretti et al. 2012 [18]	Periorbital

## Additional Points

*Institution/Department to Which the Work Should Be Attributed.* Department of Diagnostics and Therapeutics, Dentistry School of the Federal University of Bahia, address: Av. Araújo Pinho, No. 62, Canela, 40110-150 Salvador, Bahia, Brazil.
